# SLIT2 Overexpression in Periodontitis Intensifies Inflammation and Alveolar Bone Loss, Possibly via the Activation of MAPK Pathway

**DOI:** 10.3389/fcell.2020.00593

**Published:** 2020-07-14

**Authors:** Liping Wang, Jing Zheng, Janak L. Pathak, Yunxin Chen, Dongliang Liang, Luxi Yang, Haobo Sun, Mei Zhong, Lihong Wu, Li Li, Shuhua Deng, Lingyun Zheng, Yongyong Yan, Dan Hou, Lijing Wang, Linhu Ge

**Affiliations:** ^1^Guangzhou Key Laboratory of Basic and Applied Research in Oral Regenerative Medicine, Affiliated Stomatology Hospital of Guangzhou Medical University, Institute of Oral Disease, Guangzhou Medical University, Guangzhou, China; ^2^Vascular Biology Research Institute, School of Life Sciences and Biopharmaceutics, Guangdong Pharmaceutical University, Guangzhou, China

**Keywords:** SLIT2, periodontitis, alveolar bone loss, osteoclasts, MAPK signaling

## Abstract

SLIT2, a member of neuronal guidance cues, has been reported to regulate inflammation and cancer progression. Periodontitis is an oral inflammatory disease that degenerates periodontal tissue, alveolar bone and tooth. This study aims to explore the expression pattern of SLIT2 in periodontitis and its role in disease progression and bone loss. Gingival tissue of 20 periodontitis patients and 20 healthy-controls was obtained. Ligature-induced periodontitis (LIP) mice-model was developed in *Slit2-Tg* and wild-type mice. The effect of SLIT2 on inflammation, immune cell infiltration, M1 macrophage polarization, and alveolar bone loss in periodontitis was analyzed extensively. In periodontitis-affected gingival-tissue, SLIT2 expression was 4.4-fold higher compared to healthy-volunteers. LIP enhanced SLIT2 expression in mice periodontitis-affected periodontal tissue (PAPT) and blood circulation of wild-type mice by 4. 6-, and 5.0-fold, respectively. In Slit2-Tg-mice PAPT, SLIT2 expression was 1.8-fold higher compared to wild-type mice. Micro-CT and histomorphometric analysis revealed a 1.3-fold higher cement-enamel-junction to the alveolar-bone-crest (CEJ-ABC) distance and alveolar bone loss in LIP Slit2-Tg-mice compare to LIP wild-type mice. Results from RNA-sequencing, RT-qPCR, and ELISA showed a higher expression of Cxcr2, Il-18, TNFα, IL-6, and IL-1β in Slit2-Tg-mice PAPT compared to wild-type-mice. Slit2-Tg-mice PAPT showed a higher number of osteoclasts, M1 macrophages, and the upregulation of Robo1 expression. Slit2-Tg-mice PAPT showed upregulation of M1 macrophage marker CD16/32 and osteoclastogenic markers *Acp5*, *Ctsk*, and *Nfatc1*, but osteogenic markers (*Alp, Bglap*) remained unchanged. Immunohistochemistry unveiled the higher vasculature and infiltration of leucocytes and macrophages in Slit2-Tg-mice PAPT. RNA-sequencing, GO-pathway enrichment analysis, and western blot analysis revealed the activation of the MAPK signaling pathway in *Slit2*-Tg mice PAPT. In conclusion, SLIT2 overexpression in periodontitis intensifies inflammation, immune cells infiltration, M1 macrophage polarization, osteoclastogenesis, and alveolar bone loss, possibly via activation of MAPK signaling, suggesting the role of SLIT2 on exacerbation of periodontitis and alveolar bone loss.

## Introduction

SLIT2 protein, a member of neuronal guidance cues, is also expressed in extraneuronal tissues, including kidney, lung, heart, and immune cells (Yuan et al., 1999; Wu et al., 2001; Wong et al., 2002). SLIT2 is aberrantly expressed in various cancers and plays a role in the regulation of cancer cell apoptosis, cancer metastasis, tumor-associated inflammation, and tumor progression (Wang et al., 2003, 2008; Schmid et al., 2007; Avci et al., 2008; Yang et al., 2010; Zhou et al., 2011; Chang et al., 2015; Liu et al., 2018). Moreover, the SLIT2 has been reported to aberrantly regulate the inflammation in different inflammatory diseases and cell types (Zhao et al., 2014; Zhou et al., 2017; Fernando et al., 2018). SLIT2 is a secreted protein that binds to Roundabout (ROBO) receptors, i.e., ROBO1, ROBO2, ROBO3, and ROBO4. Binding of SLIT2 with specific ROBO receptor regulates particular cell functions. SLIT2/ROBO1-axis triggers proinflammatory signaling, and SLIT2/ROBO4-axis alleviates inflammation in endothelial cells during endotoxemia (Zhao et al., 2014). Periodontitis is a 6th most common chronic inflammatory disease worldwide (Kassebaum et al., 2014), affecting periodontal tissue, alveolar bone, and oral health homeostasis (Cochran, 2008; Darveau, 2010; Hajishengallis, 2014; Richards, 2014). Polymicrobial dysbiosis disrupts oral tissue homeostasis, and host immune reaction induces local and systematic inflammation in periodontitis (Darveau, 2010). Based on the existing knowledge about biological functions of SLITs in various pathophysiological conditions, (Zhao et al., 2012) had hypothesized SLIT as an effective immunotherapeutic agent in the treatment of periodontitis. However, the expression pattern of SLIT2 in periodontal tissue during periodontitis and its’ role in the pathophysiology of periodontitis is still unknown.

In periodontitis, activated proinflammatory cascade dictates the pathophysiology of the disease. Higher microvessel density (MVD) in periodontitis affected gingival tissue promotes the extravasation of inflammatory immune cells (Aspriello et al., 2009). Recruited immune cells such as macrophages and neutrophils in gingival tissue not only phagocytize the microbes but also release the proinflammatory cytokines. Enhanced macrophage infiltration in gingival tissue is frequently observed in histological images of human or animal periodontitis-affected gingival tissue sections (Yu et al., 2016). M1 macrophage polarization in periodontitis-affected gingival tissue triggers the release of proinflammatory cytokines from the immune cells and amplifies the inflammation (Yu et al., 2016; Zhou et al., 2019). Higher numbers of M1 macrophages in periodontitis-affected gingival tissue positively correlates with clinical features of chronic and aggressive periodontitis (Gonzalez et al., 2015; Yu et al., 2016; Zhou et al., 2019; Zhu et al., 2019). Neutrophil extravasates and infiltrates in periodontitis-affected gingival tissue (Zhang et al., 2020). SLIT2 has been reported to regulate neutrophil chemotaxis during lung inflammation and renal-ischemic perfusion injury (Ye et al., 2010; Chaturvedi and Robinson, 2015). However, the effect of SLIT2 on, the infiltration of leucocytes/macrophages in periodontitis-affected gingival tissue, and regulation of periodontitis-induced inflammatory cascade still need to be investigated.

Periodontitis induces periodontal tissue damage and alveolar bone resorption increasing the risk of tooth loss. Activated inflammatory cascade elevates the level of pro-inflammatory cytokines in periodontal tissue, including IL-6, IL-1β, and TNF-α. Elevated levels of pro-inflammatory cytokines in alveolar bone induce osteoclastogenesis and osteoclastogenic bone resorption (Pan et al., 2019; AlQranei and Chellaiah, 2020; Xu et al., 2020). Activation of MAPK (p38) signaling in various immune cells regulates periodontal disease progression and alveolar bone loss (Li et al., 2012; Xu et al., 2020). SLIT2 contributes to cholestatic fibrosis via activation of MAPK p38 signaling in hepatic stellate cells (Li et al., 2019). However, the effect of SLIT2 on MAPK signaling in periodontal immune cells during periodontitis is still a mystery.

In this study, we aimed to analyze the expression pattern of SLIT2 in periodontitis-affected gingival tissue, and the role of SLIT2 on the pathophysiology of periodontitis. We analyzed SLIT2 expression in the human periodontitis-affected gingival tissue and ligature-induced periodontitis (LIP)-affected mice periodontal tissue. The effect of SLIT2 on the infiltration of immune cells, M1 macrophage polarization, osteoclastogenesis, and alveolar bone loss was extensively analyzed in *Slit2*-Transgenic (Tg) periodontitis mice. Our study elucidated SLIT2 overexpression in human and mice periodontitis-affected tissue. Moreover, SLIT2 overexpression intensifies the infiltration of leucocytes/macrophages, inflammation, M1 macrophage polarization, osteoclastogenesis, alveolar bone loss, and activation of P-P38 signaling in periodontitis.

## Materials and Methods

### Collection of Human Gingival Biopsies

Human gingiva, including sulcus/pocket epithelium and underlying connective tissue, were obtained from either healthy controls (*n* = 20) or patients with periodontitis (*n* = 20) with alveolar bone loss confirmed by radiography. Patients and healthy controls clinical characteristics and demographics are summarized in Supplementary Table S1. Written informed consent was obtained from each patient. Human gingival tissues were used to analyze SLIT2 protein expression by ELISA. The Medical Ethics Committee of the Affiliated Stomatology Hospital of Guangzhou Medical University approved all protocols dealing with patients (approval number: KY2019032).

### Animals

Total of 47 wild-type C57BL/6 and 47 *Slit2* transgenic (*Slit2-Tg*) C57BL/6 mice were used in this study. Wild-type mice were purchased from Guangdong Medical Animal Experiment Center (Guangdong, China). *Slit2-Tg* mice were obtained from Prof. Lijing Wang’s lab, Vascular Biology Research Institute, School of Life Sciences and Biopharmaceutics, Guangdong Pharmaceutical University, Guangzhou, China. *Slit2-Tg* mice were generated according to the previously reported protocol (Yang et al., 2010). All the mice used were healthy and immune-normal, euthanized after their experimental periods. All studies were performed in 8-week-old male mice unless otherwise indicated. The Experimental Animal Ethics Committee of Guangzhou Medical University approved all animal care and study protocols (GY2020-004).

### Primary Bone Marrow-Derived Macrophages (BMMs) Culture

Bone marrow-derived macrophages (BMMs) were harvested from the bone marrow of six C57BL/6 mice as described previously (Spiller et al., 2016). BMMs were expanded in T75 culture flasks supplemented with Gibco RPMI 1640 medium (Life Technologies, Carlsbad, CA, United States) and 10% FBS. For the *in vitro* studies cultures were supplemented with 30 ng/ml recombinant mouse macrophage colony-stimulating factor (M-CSF, CB34, Novoprotein, Shanghai, China). Cultures were used for further mRNA and protein expression analysis.

### Development of Periodontitis in Mice

A ligature-induced periodontitis (LIP) model was developed in *Slit2-Tg* and wild-type mice, as described previously (Abe and Hajishengallis, 2013).

### Micro-CT Analysis

Micro-CT scanned the maxilla to evaluate the morphological changes in the alveolar bone. The distance from the mesial buccal cemento-enamel junction to the alveolar bone crest (CEJ-ABC) of the second molar was measured as a reference for bone loss. The level of bone resorption was calculated as described previously (Abe and Hajishengallis, 2013).

### Western Blot Analysis

Periodontitis-affected periodontal tissue (PAPT; 0.5 cm× 0.5 cm × 1.0 cm) surrounding the teeth cut from mice maxilla, including gingiva, periodontal ligament, and a part of alveolar bone, was stored at −80°C for use. Frozen periodontal tissue samples were solubilized in lysis buffer containing 10 mM of Tris–HCl, pH 7.4, 150 mM of NaCl, 1 mM of EDTA, and 1% Triton X-100 at 4°C for 20 min. The tissue lysates were subjected to centrifugation at 15000 × *g* at 4°C for 20 min. The supernatant was collected, and their protein contents were determined using Coomassie Plus Protein Assay Reagent (Pierce, Rockford, IL, United States). Cell lysate protein (50 μg) was separated by sodium dodecyl sulfate-polyacrylamide gel electrophoresis (SDS-PAGE) and transferred to polyvinylidene difluoride (PVDF) membranes. The membranes were then blocked with 5% non-fat dry milk for 1 h and incubated overnight at 4°C with primary antibodies: anti-TRAF6 (1:1000 dilution, ABclonal, Wuhan, China), anti-P38 (1:1000 dilution, ABclonal), anti-P-P38 (1:1000 dilution, Cell Signaling Technology, MA, United States), or anti-TRAP (1:1000 dilution, ABclonal). The membranes were then washed and incubated with HRP-conjugated secondary antibodies (1:5000 dilution, ABclonal) at room temperature. An enhanced chemiluminescence detection system (Thermo Scientific, MA, United States) detected the immunoreactive protein bands. Densitometry data for band intensities results were generated by analyzing the images using ImageJ software. β-actin was used as a reference housekeeping protein.

### Histological Evaluation

After scanning micro-CT images, the collected maxillae were fixed in 4% formaldehyde overnight, followed by decalcification in 10% EDTA, which was exchanged every week for 1 month. The specimens were cut in 3 mm thickness along the long axis of the molars. Next, the samples were dehydrated and embedded in paraffin blocks. Serial sections of 5 μm thickness were cut and mounted on poly-l-lysine-coated slides. Hematoxylin and eosin (H&E) staining was performed separately on consecutive tissue sections. Images were captured using a microscope. The CEJ-ABC distance between the maxillary first and second molars was measured as a net bone loss on tissue sections, using Image J software (Wang et al., 2015).

### Immunohistochemistry

Thick tissue sections (5 μm) were deparaffinized and rehydrated, followed by heat-induced epitope retrieval (Xiao et al., 2017). Methanol containing 3% H_2_O_2_ was used to block the endogenous peroxidase for 20 min. Tissue sections were then blocked with 10% bovine serum albumin (BSA) and incubated for overnight at 4°C with primary antibodies: monoclonal anti-CD34 [EP373Y], (ab81289, Abcam, Cambridge, United Kingdom), anti-CD45 (20103-1-AP, Proteintech, United States), monoclonal anti-F4/80 [BM8], (123110, BioLegend, CA, United States) or isotype controls. After washing with PBS three times, tissue sections were incubated with a corresponding goat anti-rabbit secondary antibody (SolelyBio, Beijing, China) for 30 min at 37°C. Slides were incubated with diaminobenzidine (Cell Signaling Technology, MA, United States) followed by hematoxylin counterstaining. Finally, all stained sections were dehydrated through a series of graded alcohol baths of increasing concentration, cleared in xylene, and mounted with coverslips. Stained tissue sections were visualized under a microscope (Leica, Germany), and images were captured. The visual fields between the first molar and the second molar were photographed. The number of immunostaining-positive cells was counted in the entire area of each image.

### Tartrate-Resistant Acid Phosphatase (TRAP) Staining

Tissue sections were stained using acid phosphatase (TRAP) kit (G1050, Servicebio, Wuhan, China) following the manufacturer’s instructions. TRAP stained histological tissue section were examined under microscope, and images of predefined areas were captured. TRAP-positive cells appeared bright red. TRAP-positive osteoclasts, in the alveolar bone tissue section between the first molar and the second molar, were counted.

### Real-Time qPCR

Periodontal tissue removed from mice maxillae (periodontitis-affected and control group) was homogenized in a mortar on liquid nitrogen, and total RNA was isolated using PureLink RNA Mini Kit (12183018A, Thermo Fisher Scientific, United States). RNA samples with 1.8 to 2.0 of the OD260/280 ratio were used for analysis, and total RNA reverse were transcribed using Takara PrimeScriptTM RT Master Mix in T100 Thermal Cycler (Bio-Rad, United States). The transcribed cDNA was used for RT-qPCR using Takara TB-Green Premix Ex Taq in LightCycler RT-PCR system (LC-480 II, Roche, Switzerland). GADPH was used as a reference housekeeping gene. The 2^–ΔΔCt^ method was used to analyze the relative mRNA expression levels. The primers used for RT-qPCR are listed in Supplementary Table S2.

### Protein Expression Analysis by ELISA

ELISA analyzed the protein level in human and mice periodontal tissue protein extract, mice serum, conditioned medium of mice BMMs culture, and mice BMMs cell lysates. The procedure of periodontal tissue protein extract has been described in the Western blot analysis section above. For mice serum separation, blood samples were collected from orbital venous plexus, and the serum was separated by 5 min centrifugation at 2,000 rpm. BMMs culture conditioned medium and cell lysates were prepared as follows. On the fifth day of BMMs culture, 100 ng/ml LPS was added. After 48 h of LPS treatment, conditioned medium (CM) was collected, and attached cells in the culture harvested for cell lysate preparation. SLIT2 level in human periodontal tissue lysate was analyzed by human SLIT2 ELISA kit (Cusabio, Wuhan, China) following the manufacturer’s instruction. The protein levels of IL-6, IL-1β, and TNF-a in periodontal tissue protein extract and CM were measured by mouse ELISA Standard Kit (RayBiotech, Atlanta, United States). The protein level of CD16/32 in mice periodontal tissue protein extract and the BMMs cell lysate were measured by Mouse ELISA Standard Kit (Cusabio, Wuhan, China). The protein levels of SLIT2 in mice periodontal tissue protein extract and mice serum were measured by mouse SLIT2 ELISA Kit (Cusabio, Wuhan, China) following the manufacturer’s instruction.

### Flow Cytometry Analysis

The cell suspension was prepared from mice periodontal tissue, including gingiva, periodontal ligament, and a part of alveolar bone for flow cytometry analysis (Mizraji et al., 2013). Prior to the test, cells were counted and cell viability was evaluated by the Zombie NIR^TM^ Fixable Viability Kit (423105, BioLegend, Beijing, China). Cells were stained with 1 μg of anti-F4/80 (123110, BioLegend, CA, United States) solution, monoclonal anti-CD11b [M1/70], (101206, BioLegend, CA, United States) or anti-rabbit IgG per 1 × 10^6^ cells for 30 min on ice in the dark. After two times washing with PBS, the cells were resuspended in 300 μl PBS and transferred to flow tubes. Flow cytometric analysis was performed using the FACSAria III Cell Sorter (BD Biosciences, San Jose, CA, United States).

### RNA Sequencing

The wild-type and Slit2-Tg periodontitis mice periodontal tissue samples without enzymatic treatment were used for RNA sequencing. Total RNA was extracted from periodontal tissue using the TRIzol kit (Invitrogen, Carlsbad, CA, United States), according to the manufacturer’s protocol. The RNA concentration was determined using Qubit, and RNA amount and purity of each sample was assessed with a NanoDrop spectrophotometer (NanoDrop 2000, Wilmington, DE, United States). RNA was isolated in 40 μl of DEPC water and stored in −80°C. Briefly, RNA-seq libraries were prepared by using the Illumina TruseqTM RNA sample prep Kit and were sequenced using an Illumina HiSeq. Cutadapt was used to obtain paired-end reads (Martin, 2011). The quality of the RNA-samples used for RNA-seq is presented in Supplementary Table S3. The reads were aligned with Hisat2 (v 2.1.0) to GRCm38 with default parameters (Kim et al., 2015). Only the data matched to the reference genome were used for subsequent analysis. The mapped reads of each sample were assembled using StringTie (Pertea et al., 2015). Then, the transcriptomes from all samples were merged to reconstruct a comprehensive transcriptome using perl scripts. After the final transcriptome was generated, StringTie (Pertea et al., 2015) and Ballgown (Frazee et al., 2015) was used to estimate the expression levels of all transcripts. Traditional singular enrichment analysis (SEA) (Rodriguez et al., 2016) was used for the enrichment analysis of GO terms and pathways. The enrichment *P*-value calculation was performed with Fisher’s exact test. RNA-sequence data are available at Supplementary Data S1. All raw RNA-sequencing data can be accessed from NCBI SRA database (SRA accession: PRJNA639904)^1^.

### Statistical Analyses

All data are presented as mean ± standard deviation (SD). Statistical analysis was performed using SPSS 19.0 statistical software (SPSS IBM, Armonk, NY, United States). For where appropriate (comparison of two groups only), two-tailed *t*-tests were done. In all cases, data from at least three independent experiments was used, and *P* value of less than 0.05 was considered to indicate a statistically significant difference.

## Results

### Periodontitis Amplifies SLIT2 Expression in Gingival Tissue

**FIGURE 1:**
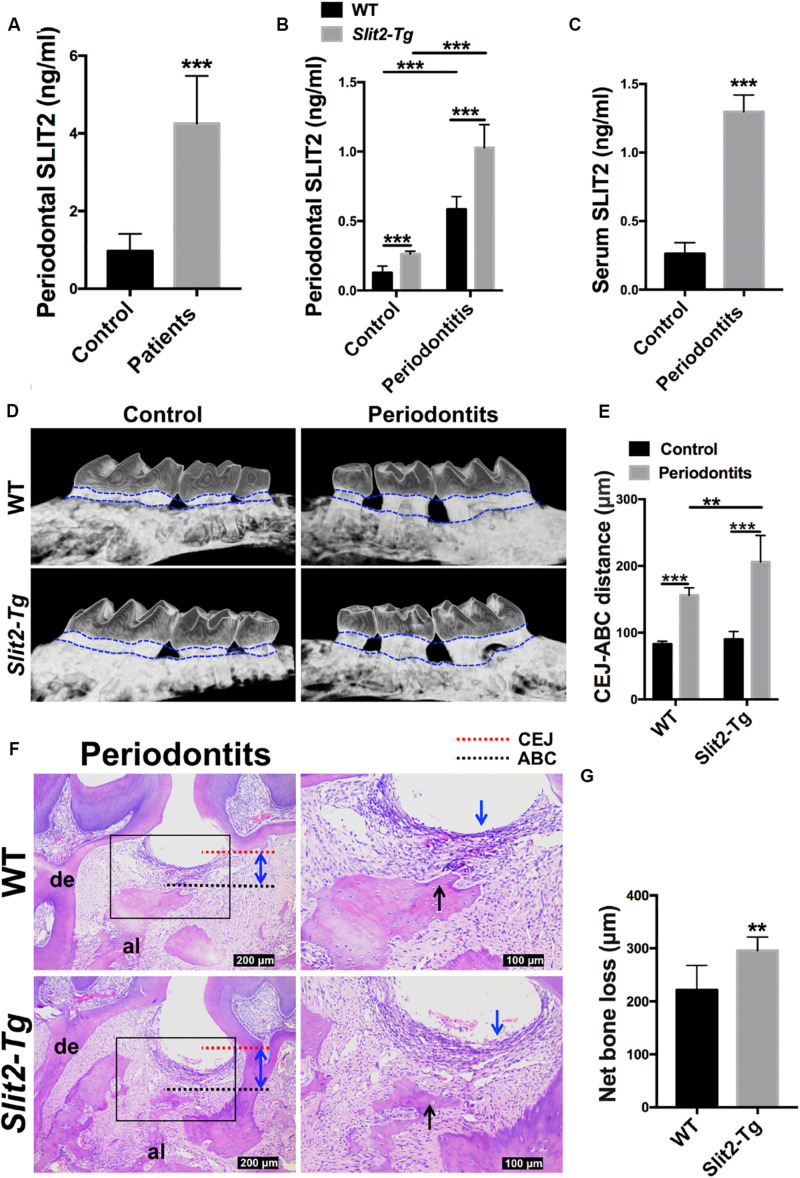
Periodontitis-induced SLIT2 aggravates periodontitis and alveolar bone loss. Expression pattern of SLIT2 protein in, **(A)** Gingival tissue of periodontitis patients and healthy individuals (*n* = 20), and **(B)** periodontal tissue of healthy and periodontitis wild-type or *Slit2-Tg* mice. **(C)** Serum level of SLIT2 protein in wild-type mice (*n* = 5). **(D)** Micro-CT images of healthy and periodontitis-affected alveolar bone with the intact tooth of wild-type and *Slit2-Tg* mice. **(E)** Quantitative data of CEJ-ABC distance analyzed from micro-CT images. **(F)** H&E stained histological images of PAPT of wild-type and *Slit2-Tg* mice. **(G)** Quantitative data of alveolar bone loss around the periodontitis-affected tooth analyzed from histological images. Data are presented as mean ± SD (*n* = 6). The significant difference among the groups, *P < 0.05, **P < 0.01, ***P < 0.001. CEJ: cement-enamel junction, ABC: alveolar bone crest, Red dot line: CEJ level, Black dot line: ABC level, al: alveolar bone, de: dentine, blue arrow: inflammatory infiltration of the gingival epithelium, black arrow: alveolar bone resorption, double arrowhead (blue): CEJ-ABC distance, and WT: wild-type.

First, we examined the SLIT2 expression in human gingival tissues of 20 periodontitis patients and 20 age and gender-matched healthy individuals. The SLIT2 protein level in periodontitis-affected human gingival tissue was 4.4-fold higher compared to healthy gingival tissue (Figure 1A). The SLIT2 protein level in the periodontal tissue of *Slit2-*Tg mice was 2.1-fold higher compared to wild-type mice (Figure 1B). The SLIT2 protein expression in wild-type mice was upregulated by 4.6-fold compared to healthy periodontal tissue (Figure 1B). Similarly, a 3.9-fold higher expression of SLIT2 protein was observed in *Slit2-Tg* mice PAPT compared to healthy periodontal tissue (Figure 1B). *Slit2-Tg* mice PAPT showed a 1.8-fold higher expression of SLIT2 protein in comparison to wild-type mice PAPT (Figure 1B). Additionally, the serum of wild-type periodontitis mice showed 5.0-fold higher expression of SLIT2 protein compared to wild-type healthy mice (Figure 1C). Our results indicate the upregulation of SLIT2 expression in gingival tissue during periodontitis in human and in PAPT of wild-type mice or *Slit2-Tg* mice. Moreover, periodontitis condition upregulates the level of serum SLIT2 in wild-type mice showing the systemic effect of periodontitis.

### Higher Expression of SLIT2 Intensifies Periodontitis and Alveolar Bone Loss

We performed micro-CT scanning to analyze the effect of LIP on alveolar bone loss. Intact periodontal tissue and normal alveolar bone were observed in healthy wild-type and *Slit2-*Tg mice (Figure 1D). Loss of periodontal tissue and alveolar bone was observed in LIP wild-type and *Slit2-Tg* mice (Figure 1D). The higher CEJ-ABC distance indicates the higher effect of periodontitis on periodontal tissue damage and alveolar bone loss. CEJ-ABC distance in periodontitis wild-type mice was 1.9-fold higher compared to healthy wild-type mice (Figure 1E). Similarly, CEJ-ABC distance in periodontitis *Slit2-Tg* mice was 2.3-fold higher compared to healthy *Slit2-Tg* mice (Figure 1E). *Slit2-Tg* periodontitis mice showed a 1.3-fold higher CEJ-ABC distance compared to wild-type periodontitis mice (Figure 1E). This finding was corroborated by the histological images as well (Figure 1F). *Slit2-*Tg mice periodontitis exhibited more bone loss and bone destruction in the alveolar crest height than in wild-type mice periodontitis (Figures 1D,E). This effect was further corroborated by the histological images (Figure 1F). The histological images showed the infiltration of inflammatory immune cells in gingival epithelial and alveolar bone resorption (Figure 1F). Histomorphometric analysis showed 1.3-fold higher periodontitis-induced alveolar bone loss in *Slit2-Tg* mice compared to wild-type mice (Figure 1G). Our results indicate that the overexpressed SLIT2 in periodontitis aggravates the consequences of the disease, such as the destruction of periodontal tissue and alveolar bone loss.

### SLIT2 Overexpression Amplifies Osteoclastogenesis and Inflammation in Periodontitis Milieu

**FIGURE 2:**
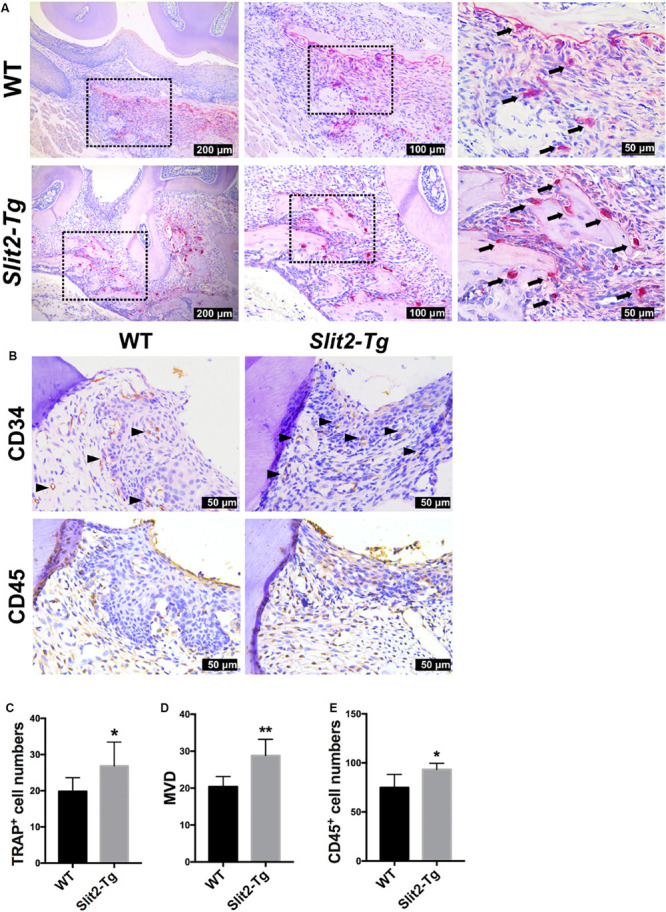
SLIT2 overexpression augments pathological changes in both periodontal milieu soft and bone tissues during periodontitis. **(A)** Representative histological images of PAPT sections showing TRAP+ osteoclasts. **(B)** Representative CD34 and CD45 immunohistochemistry images of PAPT sections. **(C)** Quantitative analysis of osteoclasts from TRAP-stained histological tissue sections (*n* = 6). **(D)** Quantification of micro-vessel density from CD34 immunohistochemistry images (*n* = 5). **(E)** Quantification of CD45 expressing cells from CD45 immunohistochemistry images (*n* = 6). The significant difference among the groups, *P < 0.05, **P < 0.01. Black arrow: TRAP+ multinucleated osteoclasts. Black triangle arrowhead: microvessel. WT: wild-type.

Inflammatory cytokines promote osteoclastogenesis, and higher osteoclastogenesis is associated with higher bone loss. In this study, we analyzed the role of the higher SLIT2 expression in periodontitis on osteoclastogenesis. More numbers of TRAP-positive osteoclasts were visualized in the PAPT section of *Slit2-Tg* mice compared to wild type mice (Figure 2A). A higher degree of vasculature in gingival tissue aggravates periodontitis. In this study, we performed CD34 immunostaining in PAPT to detect microvasculatures. Higher CD34 expression was observed in PAPT *of Slit2-Tg* mice compared to wild-type mice (Figure 2B). Quantitative analysis showed a 1.4-fold higher number of osteoclasts in PAPT in *Slit2-Tg* mice compared to wild-type mice (Figure 2C). Quantitative analysis revealed 1.4-fold higher microvessel density (MVD) in PAPT of *Slit2-Tg* mice compared to wild-type mice (Figure 2D).

Similarly, we performed immunohistochemical staining for leukocyte specific antigen, CD45, in gingival tissue sections to analyze the effect of SLIT2 overexpression on leucocytes infiltration in PAPT. More numbers of CD45 positive cells were observed in periodontitis-affected gingival tissue of *Slit2-Tg* mice compared to wild-type mice (Figure 2B). Quantitative analysis results revealed a 1.2-fold higher number of CD45-positive cells in PAPT of *Slit2-Tg* mice compared to wild-type mice (Figure 2E). This indicates that the higher degree of leucocyte infiltration in PAPT of *Slit2-Tg* mice, possibly amplify periodontal inflammation.

**FIGURE 3:**
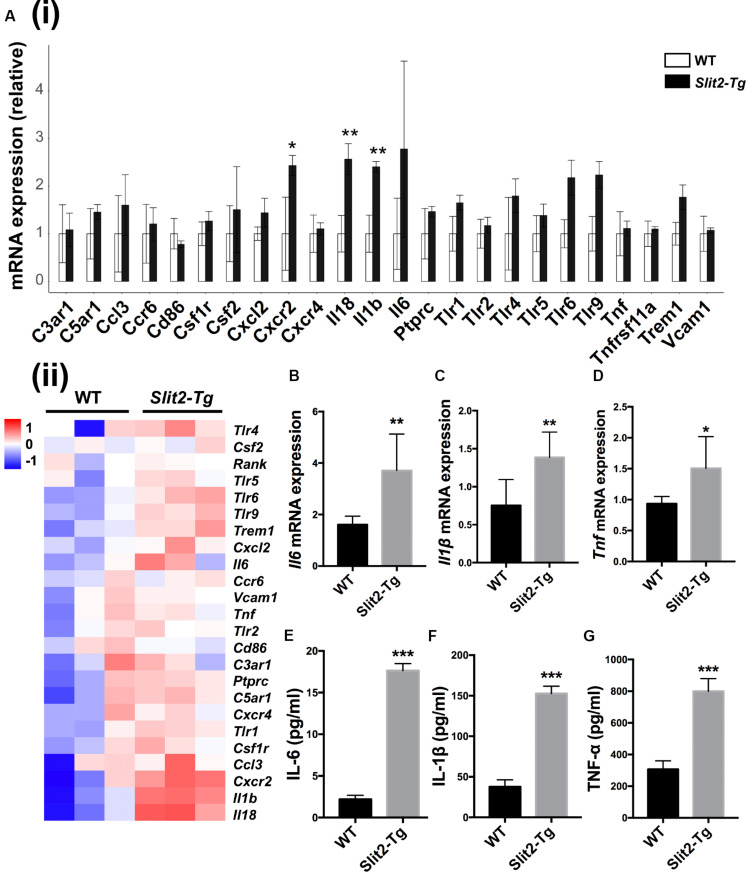
SLIT2 overexpression triggers up-regulation of periodontal inflammatory factors. **(A (i))** Relative mRNA expression, and **(A (ii))** Heat map showing the differential expression pattern of inflammatory markers in PAPT analyzed by RNA sequencing (*n* = 3). Relative mRNA expression of **(B)** *Il6*, **(C)** *Il1*β, and **(D)** *Tnfα* in periodontitis-affected tissue analyzed by RT-qPCR. Protein expression of, **(E)** IL-6, **(F)** IL-1β, and **(G)** TNF-α in periodontitis-affected tissue analyzed by ELISA. Data are presented as mean ± SD (*n* = 6). Data are presented as mean ± SD. The significant difference among the groups, *P < 0.05, **P < 0.01, ***P < 0.001. Red color intensity indicates upregulation, and blue color intensity indicates the downregulation of gene expression.

RNA sequencing (RNA-seq) of mice PAPT was performed to explore the underlying mechanisms of the regulatory effects of SLIT2 on periodontitis. Among 24 common inflammatory factors, *Cxcr2*, *Il18*, and *Il1*β were differentially upregulated by more than 2-fold in PAPT of *Slit2-Tg* mice compared to wild-type mice (Figure 3A (i)). The heat map corroborated the differential upregulation of *Cxcr2*, *Il18*, and *Il1*β mRNA in PAPT of *Slit2-Tg* mice compared to wild-type mice (Figure 3A (ii)). Highly expressed pro-inflammatory cytokines in periodontal tissue abrogate periodontitis. RT-qPCR results showed 2. 3-, 1. 8-, and 1.6-fold higher expression of *Il6* (Figure 3B), *Il1*β (Figure 3C), and *Tnf* (Figure 3D), respectively, in PAPT of *Slit2-Tg* mice compared to wild-type mice. Protein level expression of IL-6 (Figure 3E), IL-1β (Figure 3F), and TNF-α (Figure 3G) were 8-, 4- and 2.6-fold higher, respectively, in PAPT of *Slit2-Tg* mice compared to wild-type mice. This result indicates that the SLIT2-overexpression intensifies the inflammation in periodontitis.

**FIGURE 4:**
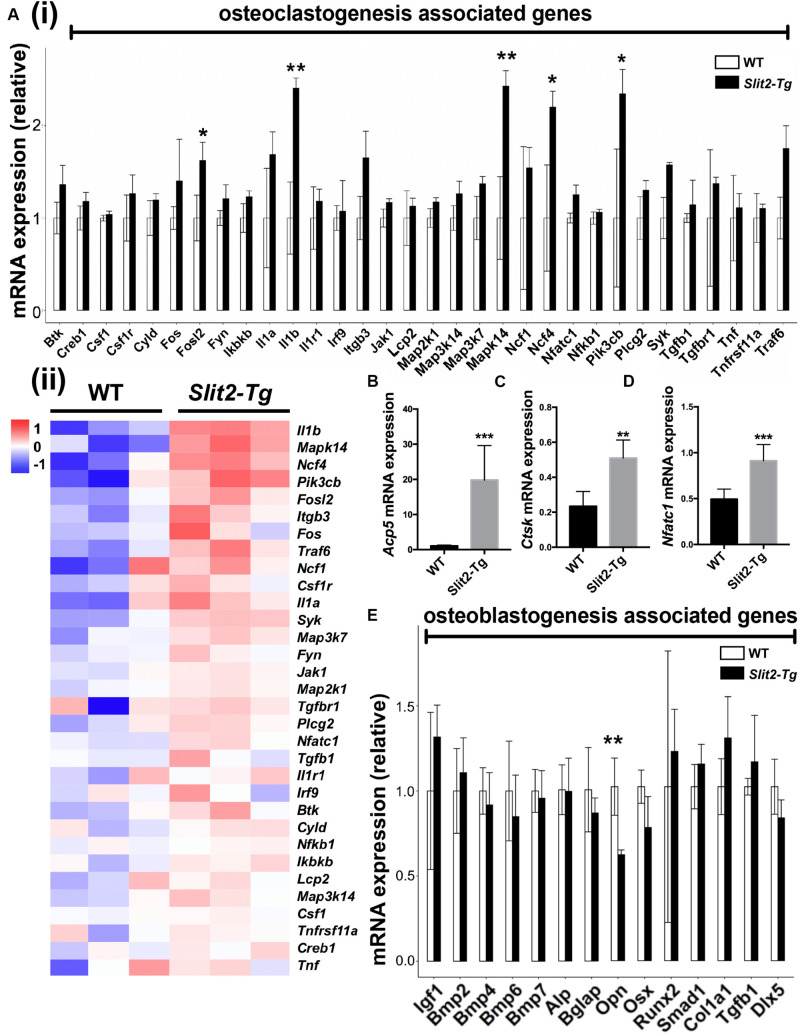
SLIT2 overexpression activates osteoclastogenesis-related signaling pathways in periodontitis. **(A (i))** Relative mRNA expression, and **(A (ii))** Heat map showing the differential expression pattern of osteoclastogenesis related genes analyzed by RNA-seq (*n* = 3). Relative mRNA expression of **(B)** *Acp5*, **(C)** *Ctsk*, and **(D)** *Nfatc1* in periodontal tissue analyzed by RT-qPCR (*n* = 6). **(E)** Relative mRNA expression of osteoblastogenesis related genes analyzed by RNA sequencing (*n* = 3). Data are presented as mean ± SD. The significant difference among the groups, *P < 0.05, **P < 0.01, ***P < 0.001. WT: wild-type.

We further analyzed the differential expression of 32 osteoclastogenesis related genes in PAPT by RAN-seq. The expression of *Fosl2, Il1β, Mapk14, Ncf4*, and *Pik3cb*, was differentially upregulated in PAPT of *Slit2-Tg* mice compared to wild-type mice (Figures 4A (i),(ii)). We further analyzed the mRNA expression of the key osteoclastogenesis regulators (*Acp5, Ctsk*, and *Nfatc1*) in PAPT by RT-qPCR. Notably, the expression of *Acp5, Ctsk*, and *Nfatc1* in PAPT of *Slit2-Tg* mice was 18. 4-, 2. 2-, and 1.8-fold higher, respectively, compared to wild-type mice (Figures 4B–D). Additionally, the expressions of the majority of osteoblastogenesis associated genes were not altered in PAPT of *Slit2-Tg* mice compared to wild-type mice. (Figure 4E and Supplementary Figure S1). These results indicate that the SLIT2 overexpression mainly triggers inflammation-induced osteoclastogenesis but might not affect the osteoblast function during periodontitis.

### SLIT2 Overexpression Enhances the Infiltration of Macrophages and M1 Macrophage Polarization in Periodontitis

**FIGURE 5:**
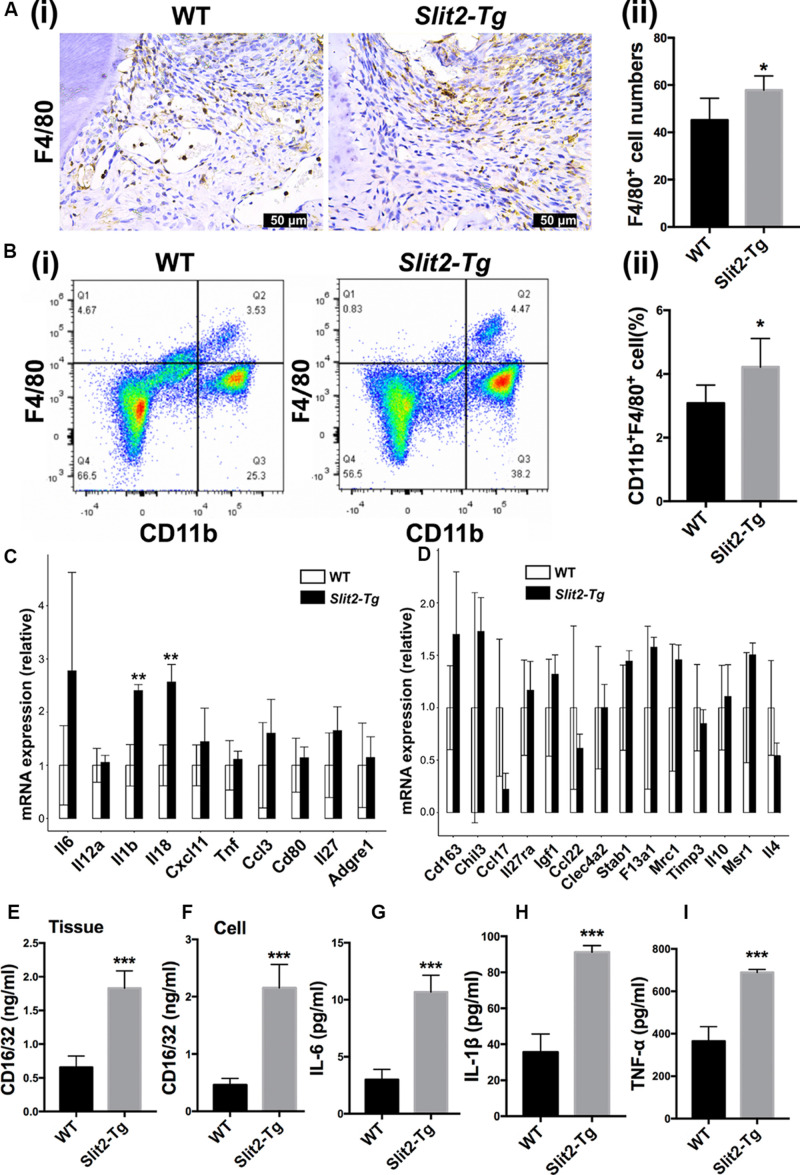
SLIT2 overexpression induces M1 macrophage polarization in PAPT. **(A (i))** Representative F4/80 immunohistochemistry images of the PAPT section. **(A (ii))** Quantification of F4/80 expressing cells in a PAPT section from immunohistochemistry images (*n* = 6). **(B (i))** Flow cytometry analysis of F4/80 positive relative frequency of gated populations from PAPT. **(B (ii))** Quantitative analysis of F4/80+ flow cytometry data (*n* = 5). Relative mRNA expression of **(C)** M1, and **(D)** M2 macrophage polarization related genes analyzed by RNA sequencing (*n* = 3). **(E)** Protein level expression of CD16/32 in PAPT (*n* = 6). Protein level expression of, **(F)** CD16/32 in cell lysates of LPS-treated BMMs. Protein expression of **(G)** IL-6, **(H)** IL-1β, and **(I)** TNF-α in conditioned medium of LPS-treated BMMs (*n* = 4). Data are presented as mean ± SD. The significant difference among the groups, *P < 0.05, **P < 0.01, ***P < 0.001.

More numbers of macrophages (F4/80 positive cells) were observed in the PAPT section of *Slit2-Tg* mice compared to wild-type mice (Figure 5A (i)). Quantitative analysis of infiltrated macrophages from immunohistochemistry images showed 1.3-fold higher numbers of macrophages in PAPT of *Slit2-Tg* mice compared to wild-type mice (Figure 5A (ii)). This finding was further verified by the data from flow cytometry analysis (Figures 5B (i),(ii)). Flow cytometry analysis revealed 1.4-fold higher numbers of CD11b+F4/80+ cells in the PAPT of *Slit2-Tg* mice compared to wild-type mice (Figure 5B (ii)). Prior to the test, cell viability was evaluated and above 95% viability of cells was ensured (data not shown). This finding indicates that the SLIT2 overexpression in periodontitis facilitates the infiltration of macrophages in periodontal tissue.

Proinflammatory cytokines trigger the M1 macrophage polarization. The relative expression of *Il1*β and *Il8* was upregulated in *Slit2-Tg* PAPT (Figure 5C). However, the expression of M2 macrophage polarization related cytokines (such as *Il10* and *Il4*) remained the same in *Slit2-Tg* PAPT and wild-type mice (Figure 5D). The protein level of CD16/32, a marker of M1 macrophage polarization, was upregulated (2.8-fold) in PAPT of *Slit2-Tg* mice compared to wild-type mice (Figure 5E). Similarly, under inflammatory condition (LPS treatment), cell lysate from *in vitro* cultured macrophages of *Slit2-Tg* mice expressed 4.7-fold higher CD16/32 compared to wild-type mice (Figure 5F). Similarly, inflammatory condition upregulated IL-6, IL-1β, and TNF-α expression in *Slit2-Tg* mice macrophages by 3. 6-, 2. 6-, and 1.9-fold, respectively, compared to wild-type mice macrophages (Figures 5G–I). This result indicates that SLIT2 overexpression amplifies macrophage infiltration and M1 macrophage polarization in PAPT.

### SLIT2 Overexpression Upregulates Its Receptor ROBO1

**FIGURE 6:**
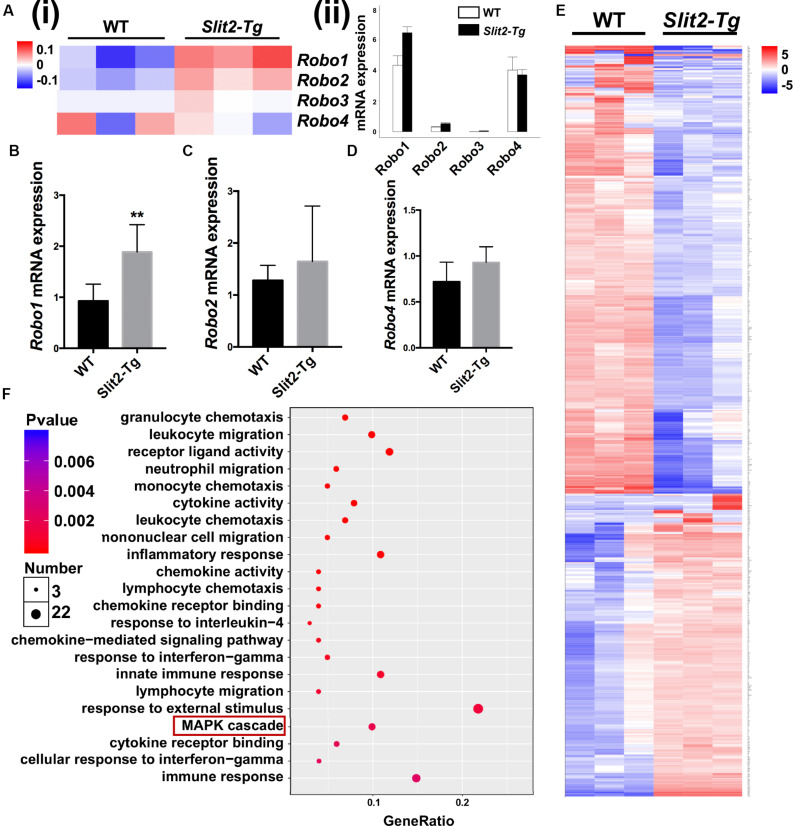
SLIT2/ROBO1 signaling possibly regulates the SLIT2-induced inflammation in periodontitis. **(A (i))** Heat map the differential expression of Slit2 receptors *Robo1-4*, and **(A (ii))** Relative mRNA expression of *Robo1*, *Robo2*, *Robo3*, and *Robo4* in PAPT analyzed by RNA sequencing (*n* = 3). Relative mRNA expression of, **(B)** *Robo1*, **(C)** *Robo2*, and **(D)** *Robo4* in PAPT analyzed by RT-qPCR (*n* = 6). **(E)** Heat map of differentially expressed 134 mRNAs in PAPT of *Slit2-Tg* mice to wild-type mice (fdr < 0.05,| FC| > 2). **(F)** GO pathway enrichment analysis. Data are presented as mean ± SD. The significant difference among the groups, *P < 0.05, **P < 0.01.

ROBO1, ROBO2, ROBO3, and ROBO4 are 4 receptors of SLIT2 ligand. In RNA-seq heat map, *Robo1* and *Robo2* gene expression were differentially upregulated, and *Robo3* and *Robo4* remained unchanged in *Slit2-Tg* mice PAPT (Figures 6A–D). Quantification of RNA-seq data showed 1.5-fold, and RT-qPCR analysis showed a 2.0-fold higher expression of Robo1 in PAPT of *Slit2-Tg* mice compared to wild type mice (Figure 6A (ii)). However, the expression of other receptors remained unchanged. This result indicates the possible role of the SLIT2/ROBO1 axis on SLIT2-mediated effects in periodontitis.

### Activated MAPK Signaling Might Regulates the SLIT2-Mediated Abrogation of Periodontitis

**FIGURE 7:**
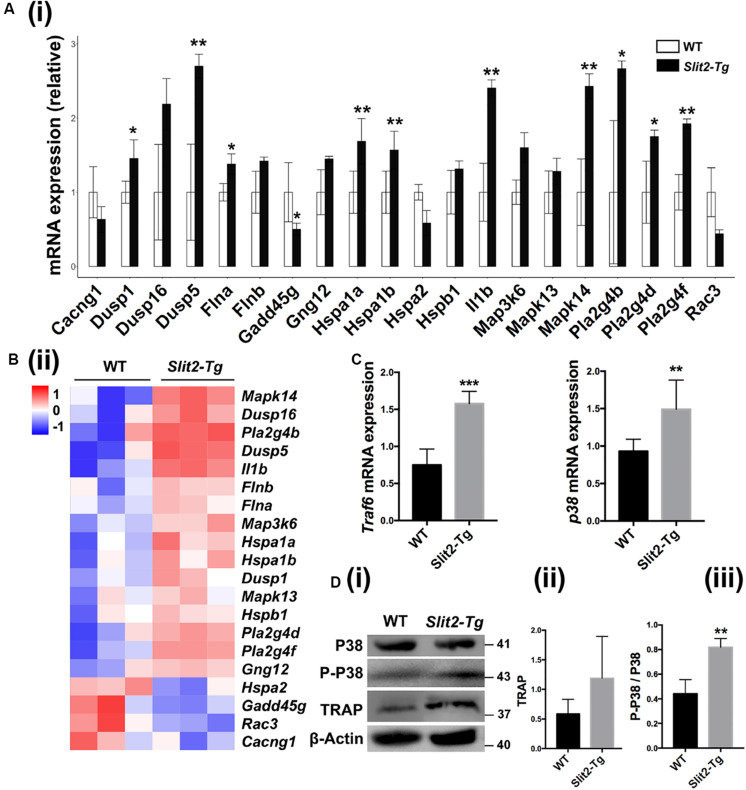
SLIT2 overexpression activates MAPK signaling pathway in periodontitis milieu. **(A (i))** Relative mRNA expression (*n* = 3), and **(A (ii))** Heat map of the differential expression pattern of MAPK signaling pathway-related genes in PAPT analyzed by RNA sequencing. Relative mRNA expression of **(B)** *Traf6*, and **(C)** *p38* in PAPT analyzed by RT-qPCR (*n* = 6). **(D (i))** Representative western blot images of MAPK signaling related proteins. **(D (ii), (iii))** Quantitative analysis of TRAP and P-P38 (*n* = 4). Data are presented as mean ± SD. The significant difference among the groups, *P < 0.05, **P < 0.01, ***P < 0.001.

RNA sequencing data detected the expression of total of 53801 genes in mice PAPT (Supplementary Data S1). Among those, 134 genes were differentially expressed in *Slit2-Tg* mice. Among 134 genes, 90 were downregulated, and 44 were upregulated (Figure 6E). GO pathway enrichment analysis showed the significant changes in expression pattern genes associated with the 22 key signaling pathways related to inflammation, vital cell functions, and others (Figure 6F). We further analyzed the expression pattern of various genes associated with the MAPK pathway. RNA-seq data analysis showed a higher expression of *Dusp1, Dusp5, Flna, Hspa1a, Hspa1b, Il1β, MAPK14, Pla2g4b, Pla2g4d*, and *Pla2g4f* in *Slit2-Tg* mice PAPT (Figure 7A). Heat map from RNA-seq data of 20 MAPK related genes are shown in Figure 7B. Similarly, RT-qPCR result showed 2.1- and 1.6-fold higher expression of *Traf6* and *p38* in PAPT of *Slit2-Tg* mice compared to wild-type mice (Figures 7B,C). Western blot analysis showed a 2-fold higher phosphorylation p38 in PAPT of *Slit2-Tg* mice compared to wild-type mice (Figures 7D (i–iii)). Our finding indicates the possible role of activated MAPK pathway in SLIT2-mediated effects on periodontitis.

## Discussion

**FIGURE 8:**
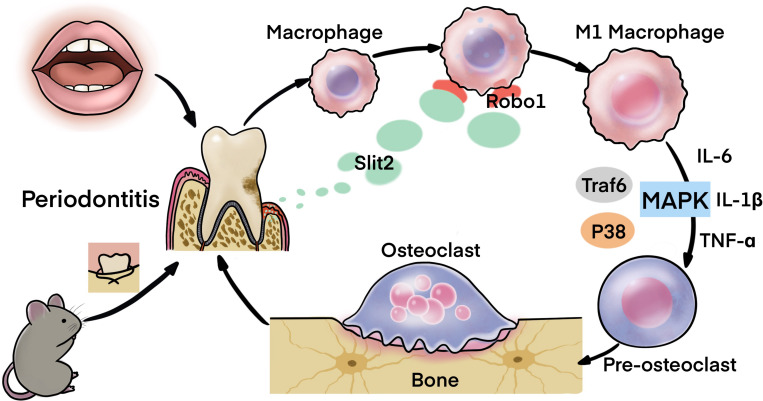
Scheme showing the role of SLIT2 overexpression in pathophysiology of periodontitis.

Our previous study reported that the higher expression of SLIT2 in cancer tissue of colorectal carcinoma promotes tumor metastasis (Yao et al., 2019). In this study, we found evident upregulation of SLIT2 in periodontitis-affected human gingival tissue, and PAPT of mice, as well as in the serum of periodontitis mice. SLIT2 overexpression did not initiate the periodontitis itself. However, SLIT2 overexpression aggravated the periodontitis and periodontitis-induced alveolar bone loss. SLIT2 intensified the inflammation in PAPT as indicated by higher MVD, and infiltration of leucocytes/macrophages in periodontitis *Slit2*-Tg mice compared to wild-type mice. Expression of proinflammatory markers Cxcr2, IL-18, IL-6, IL-1β, and TNFα were upregulated in PAPT of Slit2-Tg mice. Similarly, PAPT of *Slit2*-Tg mice showed upregulation of osteoclastogenic markers, higher numbers of osteoclasts, and M1 macrophage polarization. SLIT2/ROBO1 axis has been reported to induce proinflammatory properties (Zhao et al., 2014; Yao et al., 2019). ROBO1 receptor was upregulated in PAPT of *Slit2*-Tg mice compared to wild type mice. MAPK signaling is a key regulator of inflammation and osteoclastogenesis (Li et al., 2012; Xu et al., 2020). Expression of MAPK signaling related factors MAPK14 and p-p38 was upregulated in the PAPT of *Slit2*-Tg mice. For the first time, this study reported that the SLIT2 overexpression in periodontal tissue intensifies the periodontitis progression and alveolar bone loss possibly via the activation of MAPK pathway (Figure 8).

Literature had reported the overexpression of SLIT2 in various cancers, including gastric, colorectal, and osteosarcoma (Shi et al., 2013; Zhao et al., 2018; Yao et al., 2019). However, the expression pattern of SLIT2 in the inflammatory oral disease periodontitis is still unknown. In this study, we unraveled the >4.0-fold upregulation of SLIT2 protein in periodontitis-affected tissue of human and mice compared to healthy tissue. Our recent study showed around 2.0-fold upregulation of SLIT serum levels in colorectal carcinoma patients (Yao et al., 2019). In the present study, serum SLIT2 level in periodontitis mice was upregulated by 5.0-fold compared to healthy mice indicating systemic upregulation. Although periodontitis is an oral inflammatory disease, it has shown various systemic effects (Papapanou, 2015). This result suggests the possible role of overexpressed SLIT2 on the systemic effects of periodontitis. SLIT2 upregulated pathological condition of hepatocellular carcinoma shows a higher expression of ROBO1. Among the 4 ROBO receptors, ROBO1 was robustly upregulated in PAPT (Avci et al., 2008). Moreover, SLIT2/ROBO4 axis has reported to exert anti-inflammatory signaling and SLIT2/ROBO1 has axis been reported to exert proinflammatory signaling (Zhao et al., 2014). In this study, we found that both SLIT2 and ROBO1 were upregulated in periodontitis, suggesting the possible role of SLIT2/ROBO1 axis on escalation of inflammation and disease progression in periodontitis.

SLIT2 promotes angiogenesis (Li et al., 2015), and the migration of specific cancer cells (Zhao et al., 2016; Yao et al., 2019). SLIT2 has been reported to inhibit neutrophil migration, but enhance eosinophil chemotaxis (Tole et al., 2009; Ye et al., 2010). In this study, SLIT2 increased the MVD and leucocyte/macrophage infiltration in PAPT. Pilling et al. (2019) recently reported the SLIT2 isoform-specific neutrophil chemorepellent and chemoattractant functions. The ∼140-kDa N-terminal Slit2 fragment (Slit2-N) enhances, and the ∼110-kDa N-terminal Slit2 fragment (Slit2-S) inhibits neutrophil migration via binding with same ROBO1 receptor. The neutrophil and macrophage chemoattractant properties of SLIT2 in periodontitis might be the act of Slit2-N fragment. However, further studies illustrating the expression pattern of Slit2-N and Slit2-S and their function on neutrophil/macrophage migration in periodontitis are needed to support this hypothesis.

Disturbed host immune reaction elevates the levels of proinflammatory cytokines such as IL-8, IL-6, IL-1β, and TNF-α in PAPT, creating inflammatory milieu (Pan et al., 2019). These cytokines trigger M1 macrophage polarization, osteoclastogenesis, and immune cell infiltration that escalate inflammation and disease progression in a vicious cycle manner. Overexpression of SLIT2 enhanced the expression of proinflammatory cytokines IL-18, IL-6, IL-1β, and TNF-α. Moreover, PAPT of *Slit2*-Tg mice showed a higher degree of M1 macrophage polarization. Higher numbers of leucocytes and macrophages were infiltrated in PAPT of *Slit2*-Tg mice. Upregulation of osteoclastogenic markers *Acp5, Ctsk* and *Nfatc1*, and higher numbers of osteoclasts were observed in PAPT of *Slit2*-Tg mice. We did not witness the direct effect of SLIT2 on osteoblast function during periodontitis. SLIT2 has been reported to inhibit osteoclastogenesis and osteoblast differentiation *in vitro* (Sun et al., 2009; Park et al., 2019). The majority of pathophysiological parameters of the periodontitis were affected by SLIT2 overexpression, indicating the vital role of SLIT2 to on periodontitis progression. Our results suggest that the SLIT2-induced inflammation-mediated osteoclastogenesis might cause alveolar bone loss in periodontitis.

SLIT2 modulates MAPK signaling to regulate the various cell functions (Li et al., 2015; Xu et al., 2018; Zhao et al., 2018). MAPK signaling is a vital regulator of inflammation and pathophysiology of periodontitis (Li et al., 2012; Xu et al., 2020). GO enrichment pathway analysis of top 134 differentially expressed mRNAs in periodontitis-affected tissue of *Slit2*-Tg mice revealed the significant upregulation of MAPK signaling related genes. We further elucidated that the P-p38 was significantly upregulated in PAPT of *Slit2*-Tg mice. Along with MAPK signaling related genes, leucocytes, monocytes, and mononuclear cell chemotaxis/migration-related genes were also significantly upregulated in PAPT of *Slit2*-Tg mice. These findings further support the possible role of MAPK signaling on SLIT2-mediated effects on periodontitis.

In this study, we unraveled the higher expression of the SLIT2 in clinical gingival tissues of periodontitis patients. We further elucidated the higher SLIT2 expression in PAPT and serum of periodontitis mice. This result suggests SLIT2 as a possible diagnostic marker of periodontitis. *Slit2*-Tg periodontitis mice model unveiled the role of SLIT2 on the escalation of disease pathophysiology. This study opens the new research direction in the role of SLIT2 on the pathophysiology of inflammatory diseases. A limitation of this study is that we did not analyze which isotope of SLIT2 regulates the pathophysiology of periodontitis and whether ROBO1 is the key receptor for SLIT2-mediated effect on periodontitis. Another limitation of this study is that we did not meticulously analyze the role of MAPK in SLIT2-mediated escalation of inflammation and osteoclastogenesis. However, our ongoing study on Robo1 knockout periodontitis mice will address these limitations.

## Conclusion

SLIT2 was overexpressed during periodontitis. Overexpression of SLIT2 in periodontitis escalated inflammation, lymphocyte/macrophage infiltration, M1 macrophage polarization, osteoclastogenesis, alveolar bone loss, and disease progression. SLIT2 overexpression upregulated Robo1 and MAPK signaling related factors’ expression in PAPT during periodontitis. Our results suggest the possible role of SLIT2/ROBO1 signaling of the pathophysiology of periodontitis via activation of MAPK p38 signaling.

## Data Availability Statement

RNAseq data was uploaded to NCBI SRA database (SRA accession: PRJNA639904), https://www.ncbi.nlm.nih.gov/bioproject/PRJNA639904/.

## Ethics Statement

The studies involving human participants were reviewed and approved by The Medical Ethics Committee of the Affiliated Stomatology Hospital of Guangzhou Medical University. The patients/participants provided their written informed consent to participate in this study. The animal study was reviewed and approved by The Experimental Animal Ethics Committee of Guangzhou Medical University. Written informed consent was obtained from the individual(s) for the publication of any potentially identifiable images or data included in this article.

## Author Contributions

LG, LJW, LPW, JZ, and JP: study design. JZ, YC, DL, LY, HS, and MZ: experimental conduct. JZ, YC, DL, LY, HS, LHW, LL, SD, YY, and DH: animal work. JZ, JP, YC, DL, LY, and HS: data collection, analysis and interpretation. JZ and JP: manuscript preparation. All authors approved the final version of the manuscript.

## Conflict of Interest

The authors declare that the research was conducted in the absence of any commercial or financial relationships that could be construed as a potential conflict of interest.

## Footnotes

1 https://www.ncbi.nlm.nih.gov/bioproject/PRJNA639904/
