# Supplementary material for: SLIT2 Overexpression in Periodontitis Intensifies Inflammation and Alveolar Bone Loss, Possibly via the Activation of MAPK Pathway
Source: Front Cell Dev Biol. 2020 Jul 14;8:593. doi: 10.3389/fcell.2020.00593 (PMC7371784; doi:10.3389/fcell.2020.00593)
Supplement: Supplementary file 1 [file Data_Sheet_1.docx]

**Table S1.** Clinical characteristics and demographics of 20 healthy controls and 20 periodontitis patients included in this study

| Age/gender | Simplified oral hygiene index（OHI-S） | Bleeding index（BI） | Tooth mobility（TM） | Probing depth（PD）/mm | Gingival recession（GR）/mm | Attachment loss (AL)  /mm | furcation index（FI） | Degree of periodontitis |
| --- | --- | --- | --- | --- | --- | --- | --- | --- |
| Male/42 | 1-2 | 0-1 | 0 | 1-2 | 0 | 0 | 0 | - |
| Female/39 | 1 | 0 | 0 | 1-2 | 0 | 0 | 0 | - |
| Female/32 | 1-2 | 0 | 0 | 1-2 | 0 | 0 | 0 | - |
| Female/41 | 1 | 0-1 | 0 | 0-1 | 0 | 0 | 0 | - |
| Male/42 | 1 | 0 | 0 | 2-3 | 0 | 0 | 0 | - |
| Male/45 | 1 | 0-1 | 0 | 2 | 0 | 0 | 0 | - |
| Female/37 | 1-2 | 0 | 0 | 1-2 | 0 | 0 | 0 | - |
| Female/43 | 1-2 | 0 | 0 | 1-2 | 0 | 0 | 0 | - |
| Male/39 | 1 | 0 | 0 | 1-3 | 0 | 0 | 0 | - |
| Male/45 | 1 | 0 | 0 | 2 | 0 | 0 | 0 | - |
| Female/37 | 1 | 0-1 | 0 | 1-2 | 0 | 0 | 0 | - |
| Male/48 | 1 | 0 | 0 | 1-2 | 0 | 0 | 0 | - |
| Male/44 | 2 | 0 | 0 | 1-3 | 0 | 0 | 0 | - |
| Female/34 | 1 | 0 | 0 | 1-2 | 0 | 0 | 0 | - |
| Female/46 | 1-2 | 0-1 | 0 | 2 | 0 | 0 | 0 | - |
| Male/41 | 2 | 0 | 0 | 1-3 | 0 | 0 | 0 | - |
| Male/45 | 2 | 0 | 0 | 1-2 | 0 | 0 | 0 | - |
| Male/43 | 1 | 0-1 | 0 | 1-2 | 0 | 0 | 0 | - |
| Male/49 | 1 | 0 | 0 | 1-3 | 0 | 0 | 0 | - |
| Male/47 | 1 | 0 | 0 | 1-2 | 0 | 0 | 0 | - |
| Female/38 | 4 | 2-4 | III° | 7-8 | 2-3 | 8-11 | II-III° | +++ |
| Male/32 | 3 | 3-4 | II-III° | 7 | 2-4 | 9-11 | II-III° | +++ |
| Male/42 | 3 | 3 | III° | 8 | 3-4 | 8-12 | II-III° | +++ |
| Female/41 | 3 | 2-3 | III° | 6-7 | 3 | 9-10 | III° | +++ |
| Male/42 | 2-3 | 2-4 | III° | 6 | 2 | 8-9 | II-III° | +++ |
| Male/45 | 4 | 2-4 | III° | 7 | 2-3 | 10-12 | III° | +++ |
| Female/37 | 3 | 3-4 | III° | 6-7 | 2 | 7-9 | II-III° | +++ |
| Female/41 | 3-4 | 3 | III° | 6 | 3-4 | 9-10 | III° | +++ |
| Male/42 | 3 | 2-3 | III° | 6-8 | 2 | 8-10 | III° | +++ |
| Male/45 | 3 | 3 | II-III° | 6 | 3 | 9-11 | II-III° | +++ |
| Male/42 | 3 | 2-3 | II-III° | 7 | 2 | 9-10 | II-III° | +++ |
| Female/33 | 4 | 2-4 | III° | 6-8 | 2 | 8-10 | III° | +++ |
| Female/46 | 3 | 3 | III° | 7 | 3 | 10-11 | II-III° | +++ |
| Female/41 | 3-4 | 3-4 | III° | 6 | 2-3 | 7-8 | II-III° | +++ |
| Male/35 | 2-3 | 3-4 | II-III° | 6 | 2-4 | 7-8 | III° | +++ |
| Male/42 | 3 | 3 | II-III° | 7 | 2-3 | 9-10 | II-III° | +++ |
| Male/45 | 3 | 3-4 | III° | 6-7 | 4 | 9-12 | III° | +++ |
| Female/47 | 2-3 | 2-4 | II-III° | 5-6 | 3-4 | 8-10 | II-III° | +++ |
| Female/41 | 3 | 2-4 | II-III° | 6 | 2-3 | 7-8 | III° | +++ |
| Male/31 | 3-4 | 3 | III° | 6-8 | 3 | 9-10 | III° | +++ |

**Table S2.** Primer sequences used

| Gene | Acc. No | Primer sequence (5’ → 3’) | Product length (bp) |
| --- | --- | --- | --- |
| *Gapdh* | [NM_001289726.1](https://www.ncbi.nlm.nih.gov/entrez/viewer.fcgi?db=nucleotide&id=576080554) | F: GTGAAGGTCGGTGTGAACGG | 227 |
|  |  | R: TCCTGGAAGATGGTGATGGG |  |
| *Robo1* | [NM_019413.2](https://www.ncbi.nlm.nih.gov/entrez/viewer.fcgi?db=nucleotide&id=122114643) | F: GGTGAATCGGAGTGGTTA | 90 |
|  |  | R: CTCGTAGTTGACGCCTTT |  |
| *Robo2* | [NM_001358491.1](https://www.ncbi.nlm.nih.gov/entrez/viewer.fcgi?db=nucleotide&id=1285032866) | F: TTGGAGCAAGTTCACGGGAG | 108 |
|  |  | R: TAAGCCGCTCTGTTAGTCGG |  |
| *Robo4* | [NM_028783.4](https://www.ncbi.nlm.nih.gov/entrez/viewer.fcgi?db=nucleotide&id=1654951019) | F: TAAAGGAGAAAGGTCGTGG | 137 |
|  |  | R: TGGCGGTAGAATGAGAATAG |  |
| *Il-1β* | [NM_008361.4](https://www.ncbi.nlm.nih.gov/entrez/viewer.fcgi?db=nucleotide&id=921274059) | F: GAAATGCCACCTTTTGACAGTG | 116 |
|  |  | R: TGGATGCTCTCATCAGGACAG |  |
| *Il-6* | [NM_001314054.1](https://www.ncbi.nlm.nih.gov/entrez/viewer.fcgi?db=nucleotide&id=930945755) | F: CTGCAAGAGACTTCCATCCAG | 131 |
|  |  | R: AGTGGTATAGACAGGTCTGTTGG |  |
| *Tnf-α* | [NM_013693.3](https://www.ncbi.nlm.nih.gov/entrez/viewer.fcgi?db=nucleotide&id=518831586) | F: TGTCTCAGCCTCTTCTCATT | 153 |
|  |  | R: TGATCTGAGTGTGAGGGTCT |  |
| *Traf6* | [NM_001303273.1](https://www.ncbi.nlm.nih.gov/entrez/viewer.fcgi?db=nucleotide&id=741866085) | F: TCATTATGATCTGGACTGCCCAAC | 150 |
|  |  | R: TTATGAACAGCCTGGGCCAAC |  |
| *p38* | [NM_001357724.1](https://www.ncbi.nlm.nih.gov/entrez/viewer.fcgi?db=nucleotide&id=1274096144) | F: GATGAGCCTGTTGCTGACCCTTA | 108 |
|  |  | R: TGGTGGCACAAAGCTGATGAC |  |
| *Ctsk* | [NM_007802.4](https://www.ncbi.nlm.nih.gov/entrez/viewer.fcgi?db=nucleotide&id=530354638) | F: CAGCAGAACGGAGGCATTGA  R: CCTTTGCCGTGGCGTTATAC | 85 |
| *Nfatc1* | [NM_001164109.1](https://www.ncbi.nlm.nih.gov/entrez/viewer.fcgi?db=nucleotide&id=255759918) | F:GGTAACTCTGTCTTTCTAACCTTAAGCTC  R:GTGATGACCCCAGCATGCACCAGTCACAG | 240 |
| *Acp5* | [NM_001102405.1](https://www.ncbi.nlm.nih.gov/entrez/viewer.fcgi?db=nucleotide&id=156151434) | F: TACCTGTGTGGACATGACC  R: CAGATCCATAGTGAAACCGC | 151 |

**Table S3.** Report of RNA samples quality used for RAN-seq

| Sample | Sequences number | Bases (bp) | Error% | Concentration  (ng/μl) | | OD 260/280 | OD 260/230 | | Q20% | Q30% | GC% |
| --- | --- | --- | --- | --- | --- | --- | --- | --- | --- | --- | --- |
| WT 1 | **37551188** | **5594828782** | **0.0278** | | **318.7** | **1.97** | **1.94** | **96.77** | | **91.79** | **51.46** |
| WT 2 | **34884214** | **5199109388** | **0.0284** | | **389.7** | **2.05** | **1.72** | **96.5** | | **91.29** | **51.35** |
| WT 3 | **38345916** | **5711876862** | **0.0277** | | **408.1** | **2.04** | **1.90** | **96.8** | | **91.89** | **52.13** |
| *Slit2-Tg* 1 | **40052392** | **5963417089** | **0.0282** | | **313.8** | **2.03** | **1.85** | **96.6** | | **91.48** | **52.47** |
| *Slit2-Tg* 2 | **36421896** | **5424114754** | **0.0276** | | **366.2** | **2.01** | **1.79** | **96.83** | | **91.91** | **52.44** |
| *Slit2-Tg* 3 | **37862752** | **5636421826** | **0.028** | | **413.0** | **2.04** | **1.66** | **96.68** | | **91.6** | **52.42** |
