# Supplementary material for: SLIT2 Overexpression in Periodontitis Intensifies Inflammation and Alveolar Bone Loss, Possibly via the Activation of MAPK Pathway
Source: Front Cell Dev Biol. 2020 Jul 14;8:593. doi: 10.3389/fcell.2020.00593 (PMC7371784; doi:10.3389/fcell.2020.00593)
Supplement: Supplementary file 2 [file Image_1.pdf]

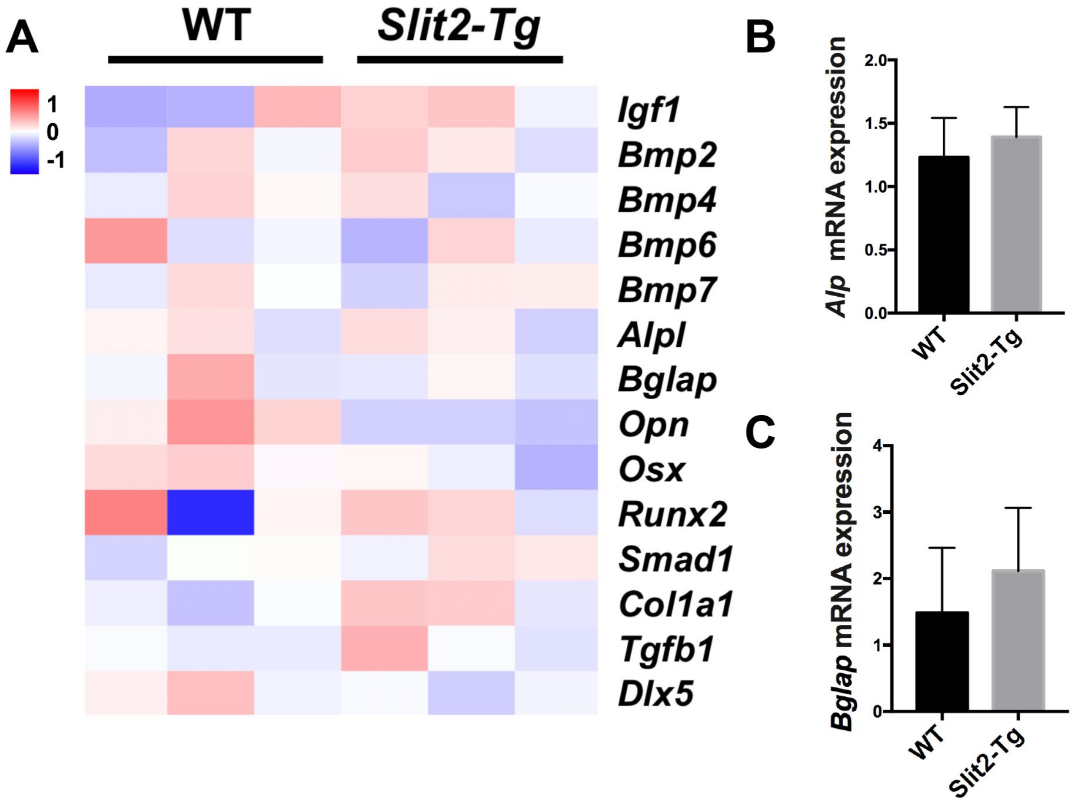

Supplementary Figure 1. (A) Heat map showing the differential expression pattern of osteoblastogenesis related genes analyzed by RNA-seq (n=3). Relative mRNA expression of (B) *Alp* and (C) *Bglap* in periodontal tissue analyzed by RT-qPCR (n=6).
