# Supplementary material for: SLIT2 Overexpression in Periodontitis Intensifies Inflammation and Alveolar Bone Loss, Possibly via the Activation of MAPK Pathway
Source: Front Cell Dev Biol. 2020 Jul 14;8:593. doi: 10.3389/fcell.2020.00593 (PMC7371784; doi:10.3389/fcell.2020.00593)
Supplement: Supplementary file 3 [file Presentation_1.pdf]

Table S1. Clinical characteristics and demographics of 20 healthy controls and 20 periodontitis patients included in this study

Age/gender	Simplified oral hygiene index（OHI-S）	Bleedi ng index（BI）	Tooth mobility（TM）	Probing depth（PD）/mm	Gingival recession（GR）/mm	Attachment loss (AL) /mm	furcation index（FI）	Degree of periodontitis
Male/42	1-2	0-1	0	1-2	0	0	0	-
Female/39	1	0	0	1-2	0	0	0	-
Female/32	1-2	0	0	1-2	0	0	0	-
Female/41	1	0-1	0	0-1	0	0	0	-
Male/42	1	0	0	2-3	0	0	0	-
Male/45	1	0-1	0	2	0	0	0	-
Female/37	1-2	0	0	1-2	0	0	0	-
Female/43	1-2	0	0	1-2	0	0	0	-
Male/39	1	0	0	1-3	0	0	0	-
Male/45	1	0	0	2	0	0	0	-
Female/37	1	0-1	0	1-2	0	0	0	-
Male/48	1	0	0	1-2	0	0	0	-
Male/44	2	0	0	1-3	0	0	0	-
Female/34	1	0	0	1-2	0	0	0	-
Female/46	1-2	0-1	0	2	0	0	0	-
Male/41	2	0	0	1-3	0	0	0	-
Male/45	2	0	0	1-2	0	0	0	-
Male/43	1	0-1	0	1-2	0	0	0	-
Male/49	1	0	0	1-3	0	0	0	-
Male/47	1	0	0	1-2	0	0	0	-
Female/38	4	2-4	III°	7-8	2-3	8-11	II-III°	+++
Male/32	3	3-4	II-III°	7	2-4	9-11	II-III°	+++
Male/42	3	3	III°	8	3-4	8-12	II-III°	+++
Female/41	3	2-3	III°	6-7	3	9-10	III°	+++
Male/42	2-3	2-4	III°	6	2	8-9	II-III°	+++
Male/45	4	2-4	III°	7	2-3	10-12	III°	+++
Female/37	3	3-4	III°	6-7	2	7-9	II-III°	+++
Female/41	3-4	3	III°	6	3-4	9-10	III°	+++
Male/42	3	2-3	III°	6-8	2	8-10	III°	+++
Male/45	3	3	II-III°	6	3	9-11	II-III°	+++
Male/42	3	2-3	II-III°	7	2	9-10	II-III°	+++
Female/33	4	2-4	III°	6-8	2	8-10	III°	+++
Female/46	3	3	III°	7	3	10-11	II-III°	+++
Female/41	3-4	3-4	III°	6	2-3	7-8	II-III°	+++
Male/35	2-3	3-4	II-III°	6	2-4	7-8	III°	+++
Male/42	3	3	II-III°	7	2-3	9-10	II-III°	+++
Male/45	3	3-4	III°	6-7	4	9-12	III°	+++
Female/47	2-3	2-4	II-III°	5-6	3-4	8-10	II-III°	+++
Female/41	3	2-4	II-III°	6	2-3	7-8	III°	+++
Male/31	3-4	3	III°	6-8	3	9-10	III°	+++

Table S2. Primer sequences used

Gene	Acc. No	Primer sequence (5' → 3')	Product length (bp)
Gapdh	NM_001289726.1	F: GTGAAGGTCGGTGTGAACGG<br>R: TCCTGGAAGATGGTGATGGG	227
Robo1	NM_019413.2	F: GGTGAATCGGAGTGGTTA<br>R: CTCGTAGTTGACGCCTTT	90
Robo2	NM_001358491.1	F: TTGGAGCAAGTTCACGGGAG<br>R: TAAGCCGCTCTGTTAGTCGG	108
Robo4	NM_028783.4	F: TAAAGGAGAAAGGTCGTGG<br>R: TGGCGGTAGAATGAGAATAG	137
Il-1β	NM_008361.4	F: GAAATGCCACCTTTTGACAGTG<br>R: TGGATGCTCTCATCAGGACAG	116
Il-6	NM_001314054.1	F: CTGCAAGAGACTTCCATCCAG<br>R: AGTGGTATAGACAGGTCTGTTGG	131
Tnf-α	NM_013693.3	F: TGTCTCAGCCTCTTCTCATT<br>R: TGATCTGAGTGTGAGGGTCT	153
Traf6	NM_001303273.1	F: TCATTATGATCTGGACTGCCCAAC<br>R: TTATGAACAGCCTGGGCCAAC	150
p38	NM_001357724.1	F: GATGAGCCTGTTGCTGACCCTTA<br>R: TGGTGGCACAAAGCTGATGAC	108
Ctsk	NM_007802.4	F: CAGCAGAACGGAGGCATTGA<br>R: CCTTTGCCGTGGCGTTATAC	85
Nfatc1	NM_001164109.1	F:GGTAACTCTGTCTTTCTAACCTTAAGCTC<br>R:GTGATGACCCCAGCATGCACCAGTCACAG	240
Acp5	NM_001102405.1	F: TACCTGTGTGGACATGACC<br>R: CAGATCCATAGTGAAACCGC	151

Table S3. Report of RNA samples quality used for RAN-seq

SAMPLE	SEQUENCES NUMBER	BASES (BP)	ERROR%	CONCENTRATION (NG/ML)	OD 260/280	OD 260/230	Q20 %	Q30%	GC%
WT 1	37551188	5594828782	0.0278	318.7	1.97	1.94	96.77	91.79	51.46
WT 2	34884214	5199109388	0.0284	389.7	2.05	1.72	96.5	91.29	51.35
WT 3	38345916	5711876862	0.0277	408.1	2.04	1.90	96.8	91.89	52.13
SLIT2-TG 1	40052392	5963417089	0.0282	313.8	2.03	1.85	96.6	91.48	52.47
SLIT2-TG 2	36421896	5424114754	0.0276	366.2	2.01	1.79	96.83	91.91	52.44
SLIT2-TG 3	37862752	5636421826	0.028	413.0	2.04	1.66	96.68	91.6	52.42
